# Healthy aging and the exercise of human rights

**DOI:** 10.1590/1518-8345.0000.3097

**Published:** 2019-01-31

**Authors:** Rosalina Aparecida Partezani Rodrigues

**Affiliations:** 1 Universidade de São Paulo, Escola de Enfermagem de Ribeirão Preto, PAHO/WHO Collaborating Centre for Nursing Research Development, Ribeirão Preto, SP, Brazil.

**Figure:**
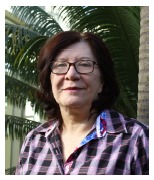

Growing old in today’s society, with a life expectancy around 70 to 80 years old, in developing and developed countries, requires a political debate, but also the implementation of key strategies for a healthy aging. In analyzing the concept of health, according to the World Health Organization^1^, the concept of functional capacity should also be associated with elderly, i.e. we should consider health-related attributes that allow people to remain and/or doing what they value, even in old age. Functional capacity results from the intrinsic capacity of the elderly, which refers to all their physical and mental capacities, combined and in interaction with the environment in which they live. Thus, the promotion and maintenance of functional capacity compose the prevailing goals for public health in the search for healthy aging. It will thus be up to the family and society to provide the elderly with the necessary resources to allow them this access considered essential to their lives, regardless of their different levels of capacity.

In short, each country should provide care to the elderly in different areas, i.e., aligning the health-care system with population needs and demands, implementing long-term services with family and community participation, integrated to essential-to-life services. Suitable environments are also required, including essential networks such as proper transport, housing, social protection, work opportunities, personal communication, as well as combating aging stereotypes and providing resources for a decent life. In addition, support services should be made available for the improvement and monitoring of a healthy aging, stimulating research on clinical interventions that consider individual aging and the problems arising from this process^1^.

Based on these international guidelines, Brazil, as a country with a life expectancy around 72 years old, presented the National Plan for Elderly Care in 1994, integrating its various Ministries to serve the elderly in its entirety^2^. After more than 20 years since the publication of the Plan, this proposal seems to be moving slowly for a variety of reasons, such as the lack of appreciation of older people by society and their lack of politicization with this proposal. Although the Statute of the Elderly^3^ affirms the Rights of the Elderly to this population, it is essential to this population that society and this group know these rights to be able to demand that they are fulfilled.

A greater effectiveness to the Statute was given with the Law 13646 of 2017, emphasizing the rights of main priority to the elderly with more than 80 years of age^4^. In this sense, the questions are, ‘How has Brazilian society treated the elderly people in different health services and social facilities?’, ‘How have nurses been paying attention to this long-lived population in the different areas of nursing care?’, ‘Are nurses prepared for a specialized care for this population?’

During the Second World Assembly on Aging in 2011^5^, the United Nations and the International Council of Nurses^6^, in their campaign “Nurses a Voice to Lead: Health is a Human Right”, posed challenges for the affirmation of human rights of people, including those of some specific groups such as the elderly. In relation to these people, it is necessary to consider: stigma and discrimination of old age and between the sexes, race, ethnicity, religion, health condition, and disabilities, as well as socio-economic aspects; poverty from malnutrition, inadequate housing, health care, and retirement; violence of all kinds, the majority in the home itself through the aggression of the family itself, and the lack of adequate services and measures to meet the specific needs of this population. In Brazil, with the enactment of the Law 13,646/2018^7^, a year of appreciation and defense of the human rights of the elderly, this debate emerges in society through a campaign of awareness of rights aiming at its effectiveness in the life of the elderly. Thus, it is the responsibility of the various social segments to implement this Law.

The right of the elderly is ensured in different legal instruments and public policies such as Plans, Statute, Laws, and other official acts. However, its implementation depends on both financial resources and health professionals to establish adequate proposals and resources from different areas for its effectiveness. Nevertheless, what is the participation of the elderly in this process? How are the elderly in the country imbued with such policies to play leading roles in society? Here are some issues to be discussed in Universities, health services, and other social instances. How do we enable the participation of this group in the definition of public policies and their co-responsibility as real subjects of “law”, considering them physically and mentally active even facing political problems, scarce health resources, and social inequality? What strategies can be used by health professionals to ensure that the human rights of the elderly are exercised in the reality of health services? These issues need to be debated in the various academic spaces and discussed, for instance, in undergraduate courses in the health area and nursing. Elderly people have their rights and must be respected as everyone in this country in order to exercise them with dignity.

This is one of the challenges of society - the implementation of policies appropriate to this age group, as well as new proposals that should emanate from these debates. The nurse specialist in gerontology has the ability to understand the process of senescence and senility in its breadth to be a protagonist of this process. Gerontological Nursing, in turn, can and should act as a potentiator of this process of social construction and emancipation!
